# Subjects with hip osteoarthritis show distinctive patterns of trunk movements during gait-a body-fixed-sensor based analysis

**DOI:** 10.1186/1743-0003-9-3

**Published:** 2012-01-20

**Authors:** Inge HF Reininga, Martin Stevens, Robert Wagenmakers, Sjoerd K Bulstra, Johan W Groothoff, Wiebren Zijlstra

**Affiliations:** 1 Department of Orthopedics, University Medical Center Groningen, University of Groningen, P.O. Box 30.001, 9700 RB, Groningen, The Netherlands; 2 Department of Orthopedics, Amphia Hospital, Breda, The Netherlands; 3 Department of Health Sciences, University Medical Center Groningen, University of Groningen, The Netherlands; 4 Center for Human Movement Sciences, University Medical Center Groningen, University of Groningen, The Netherlands

## Abstract

**Background:**

Compensatory trunk movements during gait, such as a Duchenne limp, are observed frequently in subjects with osteoarthritis of the hip, yet angular trunk movements are seldom included in clinical gait assessments. Hence, the objective of this study was to quantify compensatory trunk movements during gait in subjects with hip osteoarthritis, outside a gait laboratory, using a body-fixed-sensor based gait analysis. Frontal plane angular movements of the pelvis and thorax and spatiotemporal parameters of persons who showed a Duchenne limp during gait were compared to healthy subjects and persons without a Duchenne limp.

**Methods:**

A Body-fixed-sensor based gait analysis approach was used. Two body-fixed sensors were positioned at the dorsal side of the pelvis and on the upper thorax. Peak-to-peak frontal plane range of motion (ROM) and spatiotemporal parameters (walking speed, step length and cadence) of persons with a Duchenne limp during gait were compared to healthy subjects and persons without a Duchenne limp. Participants were instructed to walk at a self-selected low, preferred and high speed along a hospital corridor. Generalized estimating equations (GEE) analyses were used to assess group differences between persons with a Duchenne limp, without a Duchenne limp and healthy subjects.

**Results:**

Persons with a Duchenne limp showed a significantly larger thoracic ROM during walking compared to healthy subjects and to persons without a Duchenne limp. In both groups of persons with hip osteoarthritis, pelvic ROM was lower than in healthy subjects. This difference however only reached significance in persons without a Duchenne limp. The ratio of thoracic ROM relative to pelvic ROM revealed distinct differences in trunk movement patterns. Persons with hip osteoarthritis walked at a significantly lower speed compared to healthy subjects. No differences in step length and cadence were found between patients and healthy subjects, after correction for differences in walking speed.

**Conclusions:**

Distinctive patterns of frontal plane angular trunk movements during gait could be objectively quantified in healthy subjects and in persons with hip osteoarthritis using a body-fixed-sensor based gait analysis approach. Therefore, frontal plane angular trunk movements should be included in clinical gait assessments of persons with hip osteoarthritis.

## Background

Gait patterns of subjects with osteoarthritis (OA) of the hip are characterized by a decreased walking speed and step length [1-3]. Additionally, these subjects frequently show an exaggerated lateral bending of the trunk during gait, which is called a Duchenne limp [4,5]. By bending the trunk laterally towards the affected limb during the stance phase, the line of gravity working on the centre of mass (COM) of the upper body shifts closer to the affected hip joint. This decreases the mechanical demand for the hip abductor muscles by shortening the moment arm between hip and COM of the upper body, thus lowering the mechanical burden of the hip joint, resulting in pain relief [1,5]. An alternative reason for compensatory movements of the trunk during gait is that subjects with hip OA often experience weakness of the hip abductor muscles [6,7]. Consequently, they are unable to achieve stabilization of the pelvis in the frontal plane, which can be compensated for by lateral bending of the trunk [5].

Gait analysis is often used to quantify lower extremity musculoskeletal pathologies, and to evaluate progress after (surgical) interventions to improve gait function [2,8,9]. As many interventions for hip OA are aimed at regaining normal gait function [10], quantifying compensatory trunk movements in subjects with hip OA is valuable for optimal rehabilitation. Studies on compensatory trunk movements during gait of subjects with hip OA are scarce though and focus mainly on compensatory movements of the pelvis during gait [1,11], leaving the compensatory movements of the upper trunk out of sight.

Movements of body segments are usually assessed with camera-based gait analysis systems that are restricted to a laboratory setting. Therefore objective gait analysis is, until now, not feasible in clinical practice, since most clinics do not have a gait laboratory at their disposal. A disadvantage of these camera-based gait analysis systems is that they are relatively expensive, time-consuming and labor-intensive since a specialized and technically educated staff is required. As the workspace of these systems is restricted, data of only a few gait cycles can be captured. Hence, the assumption is made that data measured from only a few steps are representative of usual gait performance. Laboratory gait analysis can be viewed as inefficient and uneconomical, and its use in clinical practice is limited [12]. An alternative approach involves the use of body-fixed-sensors (BFS), which are based on the use of miniaturized and integrated motion sensors such as accelerometers and gyroscopes [13]. These BFS are relatively inexpensive, user-friendly, and lightweight, and can be carried on the body, facilitating unconstrained walking [13]. In this way data from many gait cycles can be collected outside a laboratory setting under real-life conditions. BFS-based gait systems are therefore relevant for application in clinical settings such as hospitals to monitor the effect of disease progression, (surgical) interventions, and rehabilitation on gait function. Research has shown that spatiotemporal gait parameters can be accurately measured by means of BFS [14-17]. Although a previous study which estimated hip abduction moments based on BFS demonstrated that frontal plane compensatory movements of the trunk were associated with unloading of the hip joint [18], BFS have not been applied to quantify pelvic and thoracic compensatory movements in subjects with hip OA.

The aim of the present study was therefore to quantify frontal plane compensatory movements of the trunk during gait in subjects with hip OA by means of BFS. To this end, frontal plane angular movements of the pelvis and thorax and spatiotemporal parameters of persons who showed a Duchenne limp during gait were compared to healthy subjects and persons without a Duchenne limp.

## Methods

### Participants

Sixty subjects with hip OA were included in the study. The clinical diagnosis of hip OA was made by an orthopedic surgeon of our department, based on X-ray photography and clinical signs of hip OA. These subjects were scheduled for a primary total hip arthroplasty (THA). Video recordings of gait analyses were used to determine whether persons showed a Duchenne limp during gait. Visual inspection of gait was performed according to the standard physical examination used in clinical practice [4,5]. Ten of these subjects (1 man, 9 women) were classified as persons with a clearly visible Duchenne limp. They had a mean age of 63 (SD 7) years, a mean weight of 76 (SD 10) kg and a mean height of 1.70 (SD 0.05) m. The other 50 subjects (14 men, 36 women) showed no distinct Duchenne limp. They had a mean age of 59 (SD 9) years, a mean weight of 78 (SD 12) kg and a mean height of 1.71 (SD 0.08) m. Members of several senior citizens' groups and spouses of included participants were invited to take part in the study to form the healthy control group. Thirty healthy subjects (8 men, 22 women) without clinical signs of hip OA or other conditions likely to impair gait function were included. They had a mean age of 66 (SD 6) years, a mean weight of 69 (SD 12) kg and a mean height of 1.70 (SD 0.09) m. The local Institutional Review Board approved the procedures employed in this study. All subjects gave written informed consent prior to testing.

### Apparatus

Two hybrid triaxial sensor units were used that contained gyroscopes, accelerometers and magnetometers (MTx Motion Tracker, Xsens Technologies B.V., Enschede, The Netherlands). Size of these units was 3.8 × 5.3 × 2.1 cm, weight 30 g. One of the sensor units was positioned at the dorsal side of the pelvis between the posterior superior iliac spines. The other sensor unit was positioned on the midline of the upper thorax, just below the spinal process of the seventh cervical vertebra. The sensor units were attached to the body by means of adhesive tape. The BFS were connected with a portable device (Xbus, Xsens Technologies B.V., Enschede, The Netherlands) fastened around the waist with a belt that supplied power to the BFS, sampled the BFS data, and transmitted these data in real-time to a Personal Digital Assistant (PDA) through a wireless connection (Bluetooth). With this PDA, the researcher could start and stop a measurement as well as manually place markers during data collection. All data were collected with a sample rate of 100 Hz.

### Procedure

All measurements took place in a hospital corridor, on the day of admission to the hospital for a THA. Subjects were instructed to walk a distance of 25 m back and forth on a self-selected low, preferred and high walking speed. During these measurements, markers were recorded in an additional measurement channel every time the subject passed the 2.5-m and 22.5-m point of the 25 m. Before data collection, all subjects walked the corridor on a self-selected preferred walking speed to familiarize themselves with the measurement procedure. Previous research has shown this gait analysis protocol to be reliable [17].

### Data analysis

Data were transmitted from the PDA to a PC, where the data were processed with Xsens software (MT software version 2.8.5, Xsens Technologies B.V., Enschede, The Netherlands). Next, data were further processed with Matlab (Version 7.0, The Mathworks Inc., Natick, USA). Lindemann et al. [19] recommended excluding gait data from the first 2.5 m of a walking trial in older adults to assess steady state gait, so gait data from the first and last 2.5 m of the walking trials were excluded and the middle 20 m, as identified by markers placed on the data, was used for further analysis.

For each walking trial, mean peak-to-peak amplitude of the pelvis and the thorax was determined based on 10 subsequent stride cycles. Stride cycles were selected based on initial foot contact as determined from forward pelvic accelerations [16]. The peak-to-peak frontal plane range of motion (ROM) of the thorax and the pelvis was determined by calculating the difference between the minimum and maximum angles of the segments. The ratio of thoracic ROM relative to pelvic ROM was calculated (thoracic ROM/pelvic ROM).

The spatiotemporal variables analyzed included walking speed, step length and cadence (steps/min). Mean walking speed was determined based on intermarker distance (20 m) and intermarker duration. The mean of the back-and-forth walks per instructed walking speeds were used for statistical analysis.

### Statistical analyses

Statistical analysis was done using the PASW software package (version 18, SPSS, Chicago, USA). To assess group differences between persons with a Duchenne limp (DL), without a Duchenne limp (NDL) and the healthy control group (HC), generalized estimating equations (GEE) analyses were used. Since repeated measurements (walking on low, preferred and high speed) of a subject are not independent of each other, a correction must be made for these within-subject correlations. With GEE, this correction is carried out by adding a correlation structure as a covariate to the analysis. In these analyses, an exchangeable working correlation structure and robust estimation of the covariance matrix was used [20]. Additionally this analysis accurately controls for the effect of differences in walking speed and in subject characteristics such as age, body height, and body weight on the outcome variables by including these variables as covariates. To determine whether there were significant differences between the subject groups in the relationships of gait parameters with walking speed, interaction terms (group-by-walking speed interaction) were also added to the GEE-analyses. If these interaction terms were statistically significant, they were included in the GEE models. Walking speed was centered on the mean walking speed, based on all instructed walking speeds, of subjects with hip OA, 1.1 m/s, by subtracting this value from the measured walking speed. Centering allowed for a meaningful interpretation of main effects, i.e. the main effect can be interpreted as the effect of group at a walking speed of 1.1 m/s. The healthy control group was set as the reference group in the GEE models. Post hoc analyses were conducted to determine significant differences in outcome measures between DL and NDL. P-values of less than .05 were considered to be statistically significant.

## Results

### Range of Motion

**Figure 1:**
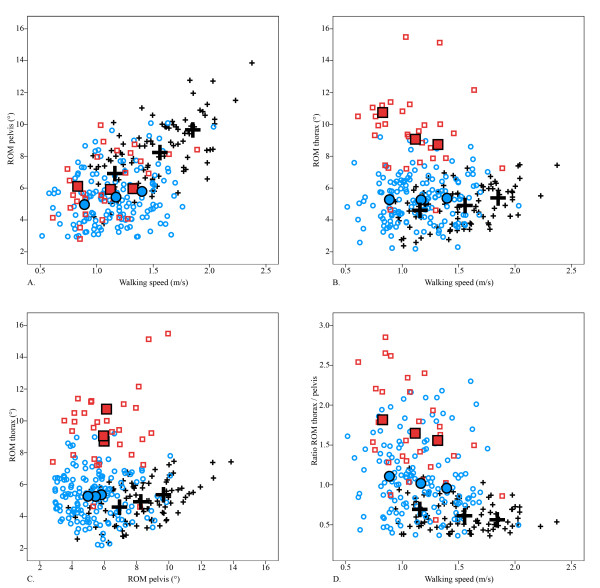
**All data are presented for low, preferred and high walking speed**. Small figures indicate individual values, large figures indicate mean values. Healthy controls (+: black); patients with a Duchenne limp (□: red); patients without a Duchenne limp (○: blue). Abbreviations: ROM, Range of Motion.

Mean ROM and ratio of the ROM of the thorax to the pelvis are presented in Figure 1 and Table 1. Table 2 shows the results of the GEE analysis of thoracic ROM, pelvic ROM and ratio of thoracic ROM to pelvic ROM.

**Table 1:** ROM and ratio of thoracic ROM to pelvic ROM

	Instructed walking speed	HC (n = 30)	NDL (n = 50)	DL (n = 10)
Pelvic ROM	Low speed	6.9 (1.5)	5.0 (1.5)	6.1 (1.8)
	Preferred speed	8.2 (1.7)	5.4 (1.6)	5.9 (2.1)
	High speed	9.7 (1.8)	5.8 (1.9)	6.0 (1.8)
Thoracic ROM	Low speed	4.6 (1.2)	5.3 (1.6)	10.7 (2.1)
	Preferred speed	4.9 (1.2)	5.3 (1.4)	9.1 (2.8)
	High speed	5.4 (1.3)	5.4 (1.4)	8.7 (2.0)
Ratio	Low speed	0.7 (0.2)	1.2 (0.5)	1.8 (0.5)
	Preferred speed	0.6 (0.2)	1.1 (0.5)	1.7 (0.6)
	High speed	0.6 (0.1)	1.0 (0.5)	1.7 (0.6)

Values are given as mean (SD). Abbreviations: ROM, Range of Motion; HC, healthy control group; NDL, patients without a Duchenne limp; DL, patients with a Duchenne limp. Thoracic and pelvic ROM are expressed in degrees (°).

**Table 2:** GEE analysis of pelvic and thoracic ROM and ratio of thoracic ROM to pelvic ROM^a^

Outcome	Group	Group effect (95% CI)	P value	Group by walking speed effect (95% CI)	P value
Pelvic ROM	HC	0^b^		0	
	NDL	-1.5 (-2.3, -0.7)	< .001	-2.4 (-3.4, -1.4)	< .001
	DL	-0.6(-1.8, 0.5)	.29	-3.0 (-4.7, -1.2)	.001
Thoracic ROM	HC	0		0	
	NDL	1.0 (0.4, 1.7)^†^	.002	-1.0 (-1.7, -0.3)^†^	.005
	DL	5.0 (3.5, 6.4)	< .001	-4.4 (-6.4, -2.4)	< .001
Ratio	HC	0		0	
	NDL	0.5 (0.3, 0.7)^†^	< .001	-0.0 (-0.2, 0.1)^†^	0.64
	DL	1.0 (0.7, 1.3)	< .001	-0.5 (-0.9, -0.1)	.007

Reference group: healthy control group.

^a ^Adjusted for walking speed, age, body height and body weight.

^b ^Set to zero because HC was used as reference group.

^† ^indicate significant difference between NDL and DL.

Abbreviations: GEE, generalized estimating equation; HC, healthy control group; NDL, patients without a Duchenne limp; DL, patients with a Duchenne limp.

Thoracic and pelvic ROM are expressed in degrees (°).

In HC, pelvic ROM showed a large increase with higher walking speed. Thoracic ROM increased slightly with higher walking speed. Overall, pelvic ROM was larger than thoracic ROM, which is reflected in a ratio lower than 1. The ratio slightly decreased with increasing speed.

In DL, pelvic ROM was slightly smaller compared to HC, though this difference was not statistically significant. Pelvic ROM remained constant with increasing walking speed. The significant (negative) group by walking speed interaction indicates that the development of pelvic ROM with increasing speed differed significantly from HC, i.e. compared to HC it increased less (2.4 degrees) when walking speed increased with 1.0 m/s (Table 2). Thoracic ROM was significantly larger in DL than in HC; at a (centered) walking speed of 1.1 m/s, the difference in thoracic ROM was 5 degrees (Table 2). Thoracic ROM decreased with higher walking speed. Thoracic ROM was larger than pelvic ROM: the ratio was larger than 1.0 at all walking speeds. At a walking speed of 1.1 m/s, the ratio of DL was 1 point higher than the ratio of HC. Furthermore, the ratio decreased significantly more (0.5 points) per 1.0 m/s increase in walking speed compared to HC, as indicated by the significant group by walking speed interaction (Table 2).

In NDL, pelvic ROM was significantly smaller and increased less with higher walking speed, compared to HC. No difference was found in pelvic ROM between NDL and DL. Thoracic ROM was slightly larger compared to HC (1 degree), but significantly smaller compared to DL. Thoracic ROM retained the same magnitude with increasing walking speed. The magnitudes of thoracic and pelvic ROM were comparable, with a ratio of around the value 1 at all walking speeds. The difference in ratio was significant between all groups (Table 2). At a (centered) walking speed of 1.1 m/s, the ratio of DL was 0.5 point higher than the ratio of HC (Table 2). There was no significant difference in the development of the ratio with increasing walking speed between NDL and HC.

### Spatiotemporal parameters

Mean walking speed, step length and cadence data are presented in Table 3. Table 4 shows the results of the GEE analysis of walking speed, step length and cadence.

**Table 3:** Walking speed, step length and cadence data

	Instructed walking speed	HC (n = 30)	NDL (n = 50)	DL (n = 10)
Walking speed	Low speed	1.2 (0.1)	0.9 (0.1)	0.8 (0.1)
	Preferred speed	1.6 (0.2)	1.2 (0.2)	1.1 (0.2)
	High speed	1.9 (0.2)	1.4 (0.2)	1.3 (0.3)
Step length	Low speed	68.6 (8.6)	60.1 (8.6)	57.3 (8.0)
	Preferred speed	79.7 (11.6)	70.0 (10.9)	65.0 (8.9)
	High speed	90.5 (11.6)	76.6 (12.8)	72.8 (11.3)
Cadence	Low speed	102.3 (9.4)	88.7 (11.4)	89.1 (8.4)
	Preferred speed	119.0 (16.4)	99.8 (15.1)	101.3 (20.2)
	High speed	123.6 (11.8)	107.8 (14.0)	110.7 (14.5)

Values are given as mean (SD). Abbreviations: HC, healthy control group; NDL, patients without a Duchenne limp; DL, patients with a Duchenne limp. Walking speed is expressed in m/s, step length in cm and cadence in steps/min.

**Table 4:** GEE analysis of walking speed, step length and cadence^a^

Outcome	Group	Group effect (95% CI)	P value
Walking speed	HC	0^b^	
	NDL	-0.4 (-0.4, -0.3)	< .001
	DL	-0.4 (-0.5, -0.3)	< .001
Step length	HC	0	
	NDL	0.4 (-2.5, 3.3)	.79
	DL	-1.0 (-5.0, 3.0)	.63
Cadence	HC	0	
	NDL	-0.1 (-4.4, 4.4)	.99
	DL	1.5 (-4.9, 8.1)	.64

Reference group: healthy control group.

^a ^Adjusted for walking speed, age, body height and body weight (walking speed: adjusted for age, body height and body weight).

^b ^Set to zero because HC was used as reference group.

Abbreviations: GEE, generalized estimating equation; HC, healthy control group; NDL, patients without a Duchenne limp; DL, patients with a Duchenne limp.Walking speed is expressed in m/s, step length in cm and cadence in steps/min.

Compared to HC, DL and NDL walked at a significantly lower speed. No differences were found in walking speed between NDL and DL. There were no significant differences in step length or cadence between the groups, after correction for walking speed.

## Discussion

The present study quantified compensatory movements of the trunk during gait in subjects with hip OA by means of BFS. Frontal plane angular movements of the pelvis and thorax and spatiotemporal parameters of persons with a Duchenne limp during gait were compared to healthy subjects and to persons without a Duchenne limp. The results showed that, over a range of instructed walking speeds, all subjects with hip OA walked at a significantly lower speed compared to healthy subjects, along with a shorter step length and lower cadence. However, after correction for walking speed, these differences in spatiotemporal parameters disappeared. Persons with a Duchenne limp showed a significantly larger thoracic ROM during walking compared to healthy subjects and to persons without a Duchenne limp. The ratio of thoracic ROM to pelvic ROM revealed distinct differences in trunk movement patterns.

Several studies reported on frontal plane ROM of the pelvis and thorax during walking on a preferred walking speed in healthy subjects of different age groups, captured with camera-based gait analysis systems [11,21-25]. Values for mean pelvic ROM ranged from 5.7 to 11.5° [11,22-25]. Values for mean ROM of the trunk ranged from 3.3-7.0° [21-25]. Our results for healthy subjects are in line with these findings.

A few studies have quantified the pelvic frontal plane ROM during walking of persons with mild hip OA, measured with camera-based gait analysis systems during overground walking on a preferred walking speed [1,11,22]. Values for mean pelvic ROM ranged from 4.0 to 6.1°. Only Thurston [22] reported a mean thoracic frontal plane ROM of 7.2°. None of these studies distinguished between persons with and without a Duchenne limp though. Our results are in line with these findings, when the results of the persons with and without a Duchenne limp are combined.

The hip abductor muscles control frontal plane pelvic movement during gait and re-establish the pelvis as a platform on which the trunk rests during the stance phase [26]. In the present study, a large pelvic ROM and a smaller thoracic ROM were observed in healthy subjects. Additionally, pelvic ROM greatly increased with higher speed, while only a slight increase in thoracic ROM was discerned. These observations indicate that with increasing walking speed the upper trunk maintains its approximately vertical orientation, while angular movements of the pelvis steadily increase. Obviously the latter requires a mounting effort by the hip abductor muscles. However, subjects with hip OA have a substantial loss of hip abductor muscle strength in the affected limb, compared to healthy age-matched controls [6,7]. Persons with a Duchenne limp showed a larger thoracic ROM, but, in contrast to those persons without a Duchenne limp, their pelvic ROM did not differ significantly from healthy controls. This finding may indicate that an excessive lateral bending of the trunk reduces the loading of the hip and hip abductor muscles, thus helping to maintain the angular movements of the pelvis. Persons with a Duchenne limp showed a decrease of thoracic ROM with higher walking speed. This may be due to the fact that with increasing speed less time is spent in single stance; thus there may be less need for compensatory movements, but also the available time to perform such an excessive lateral movement is shorter. It should be noted that persons without a Duchenne limp also showed a slightly increased thoracic ROM, but it was significantly smaller than that of persons with a Duchenne limp.

The different patterns of angular movements of the pelvis and thorax during gait can be quantified by the ratio of thoracic ROM to pelvic ROM. In healthy subjects, thoracic ROM was smaller than pelvic ROM, which is reflected in a ratio lower than 1. The ratio of persons without a Duchenne limp was around 1, that of persons with a Duchenne limp was greater than 1. The ratio of persons without a clearly visible Duchenne limp was also significantly higher compared to healthy subjects. However, in these patients, compensatory movements could not be recognized by clinical examination.

Some may consider differences in walking speed between subjects as a limitation of this study due to the confounding effects these differences might have on angular movement of the trunk and on spatiotemporal parameters. By instructing the subjects to walk at their self-chosen speed allowed each subject to walk as naturally as possible, thereby obtaining the best representation of their true (real-life) gait behavior. Furthermore, a previous reproducibility study of this gait analysis protocol has shown that, by instructing the subjects to walk at a self-chosen speed, reproducible and reliable results are obtained [17]. We therefore used statistical procedures to adjust the gait data for differences in walking speed, as recommended in the literature [27].

A decreased walking speed with shorter steps and lower cadence, as observed in this study, are typical gait adaptations in subjects with hip OA [1-3]. Reducing cadence and step length might be a compensatory strategy to lower the duration of single stance, as it results in spending a proportionally longer time in the double-support phase of the gait cycle [28]. After controlling for walking speed and several subject characteristics, our study did not find significant differences in step length or cadence between persons with hip OA and healthy subjects. By contrast, other studies reported shorter step length and an increased cadence in persons with hip OA compared to their healthy counterparts, while walking overground [11] or on a treadmill [29]. However, unlike the present study, Kubota et al. [11] did not (mathematically) control for differences in walking speed, and the results of Bejek et al. [29] may deviate from our results since spatiotemporal parameters obtained during treadmill walking may differ from those for overground walking [30].

The classification system that was used in this study to determine whether a subject showed a Duchenne limp might also be seen as a limitation of the study. In this study, persons with hip OA were classified as having a Duchenne limp by means of visual inspection of gait according to standard physical examination used in clinical practice. This is a subjective, qualitative measure. There may have been patients that use frontal plane compensatory movements of the trunk that remain unnoticed by visual inspection. Consequently, these persons might have been classified as not having a Duchenne limp. To our knowledge, there presently is no objective clinical measure to quantify a Duchenne limp. However, despite the fact that there may have been misclassifications in the non-Duchenne limp group, the results of this study were very clear. This study showed that frontal plane compensatory trunk movements, as well as spatiotemporal gait parameters, can be objectively quantified by means of a BFS-based gait analysis system. The ratio of thoracic ROM to pelvic ROM appeared to be a powerful measure to distinguish between the patterns observed in healthy subjects and in subjects with hip OA with and without a clearly visible Duchenne limp.

Until now, the primary choice of measurement to objectively monitor the effect of disease progression, (surgical) interventions, and rehabilitation on gait function is the use of a camera-based gait analysis system which is restricted to a laboratory setting. This makes it, in clinical practice, difficult to objectively quantify gait function. The easy-to-use BFS-based gait analysis system used in this study demonstrated a great potential to evaluate and objectively quantify in a clinical setting the compensatory trunk movements as well as spatiotemporal gait parameters in subjects with hip OA. However, further research is needed to determine whether the BFS-based gait analysis system can be used to assess changes in compensatory trunk movements and spatiotemporal parameters due to (surgical) treatment of hip OA.

There is a growing base of knowledge on compensatory angular trunk movements during gait in subjects with OA of the lower limb [31-33]. This has led to the development of gait retraining interventions, which use frontal plane angular trunk movement during gait to reduce joint loading of the hip and knee in persons with hip or knee OA [34,35]. Real-time biofeedback methods appeared to be effective for gait retraining [36]. Hunt et al. [34] showed that the use of biofeedback to control the amount of trunk lean during gait retraining in persons with knee OA is successful. However, they used a camera-based gait analysis system, which was bound to a gait laboratory, as a biofeedback system. Previous research has shown the feasibility of BFS as a wireless real-time auditory or visual biofeedback system during interventions to enhance balance and gait function in patients with various mobility disorders [37,38]. This application of BFS may enhance a broad implementation of biofeedback-based gait retraining interventions focused on increased frontal plane trunk movement in persons with hip and knee OA.

## Conclusions

The present study is, to our knowledge, the first to have investigated frontal plane compensatory movements of the trunk during gait of persons with hip OA by means of a body-fixed-sensor based gait analysis system. Distinctive patterns of frontal plane angular trunk movements during gait could be objectively quantified in healthy subjects and in subjects with hip OA. The ratio of thoracic ROM to pelvic ROM appeared to be a powerful measure to distinguish between the patterns observed in healthy subjects and in subjects with hip OA with and without a clearly visible Duchenne limp. No differences in spatiotemporal parameters were found, after correction for differences in walking speed. The findings of the present study suggest that frontal plane angular trunk movements should be included in clinical gait assessments of subjects with hip OA. Since this BFS-based gait analysis approach is not confined to a laboratory and is user-friendly, it is a useful method to objectively assess gait function in a clinical (outpatient) setting.

## Competing interests

The authors declare that they have no competing interests.

## Authors' contributions

IHFR, MS and WZ designed the study. IHFR carried out the data acquisition, performed the statistical analysis and drafted the manuscript. MS and WZ were involved in interpretation of results and critical revision of the manuscript. RW, SKB and JWG have also critically revised the manuscript. All authors read, edited and approved the manuscript.
